# Doing better and being better in breast cancer care: an interview with Funmi Olopade

**DOI:** 10.1242/dmm.049198

**Published:** 2021-09-22

**Authors:** Olufunmilayo I. Olopade


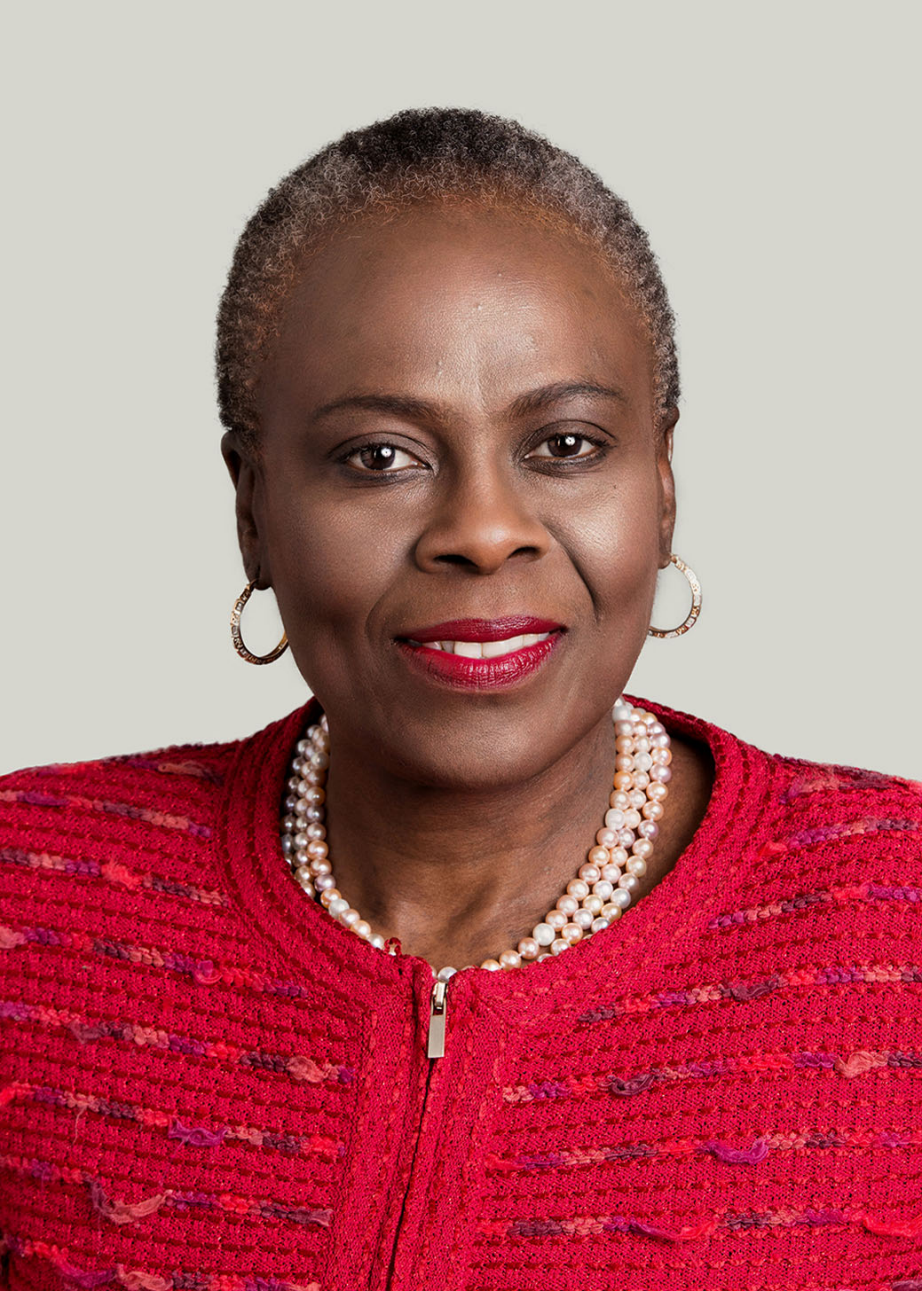


Dr Olufunmilayo (Funmi) I. Olopade, FAACR, is the Walter L. Palmer Distinguished Service Professor of Medicine and Human Genetics and Director of the Center for Clinical Cancer Genetics and Global Health at University of Chicago Medicine. She received her MD from the University of Ibadan, College of Medicine, Nigeria, and continued her training in Chicago. As a physician scientist, her research focuses on early detection, prevention and treatment of breast cancer in high-risk patients in the United States and across West Africa. Dr Olopade's seminal observations on the genetics of breast cancer in young women of African ancestry has broadened our understanding of the complexities of breast cancer causation. Throughout her career, Dr Olopade received several awards, including the Doris Duke Distinguished Clinical Scientist and Exceptional Mentor Award, a MacArthur Foundation ‘Genius’ Fellowship and the Officer of the Order of the Niger Award. She is a member of several professional societies and actively involved in many community organisations. In this interview, Funmi talks about her serendipitous research path, improving care for breast cancer patients and the heroism of women in genetics.

**You started your career as a clinician. Was there a specific moment that motivated you to go into research?**

I went to medical school in Nigeria and I didn't really have the opportunity to participate in research. But when I moved to the University of Chicago for my postgraduate training, we were expected to learn the science of oncology. I was fortunate to be in Janet Rowley's lab. She was such an inspirational female scientist who really led the field of cytogenetics [Dr Rowley was the first to identify chromosomal translocations as causes of cancer]. Just listening to her and the way she taught us made it plausible for a woman to do science and excel at it. While joining her lab was a requirement, I became immersed in the work and it was so interesting. Specializing in oncology, where there is no cure for most patients, requires a deep understanding of the science behind the field. But it was my intellectual curiosity that made me want to continue to work in the lab as part of my career. The transition was not easy but it was feasible by being in a lab of a successful investigator, as it allowed my career to evolve in a way that I think was extraordinary. We did interdisciplinary work and, because the University of Chicago has a medical campus in which the hospital, the medical school and research labs are at the same location, it was easy to go back and forth and integrate my work in the clinic and in the lab.

**In the genomics era, identifying high-risk variants and counselling carriers seems like such an obvious course, but things have not been quite as straightforward. Can you tell us a bit more?**

In 1996, when *BRCA1* was identified and the knockout phenotype was published, an interesting paper described the clinical outcomes in patients with germline *BRCA1* mutations. I was asked to write an editorial about it and, in my naïveté as a young investigator, I thought we should test everybody. The phenotype was so profound that if we identified at-risk carriers before they developed cancer, we would definitely prevent a lot of disease. As oncologists, we break bad news all the time and I believed telling someone that they are at risk was not as bad if I could help them manage that risk. It seemed so obvious to me.

“I absolutely fought against the genetics community, arguing that this [BRCA1-driven breast cancer] is not like Huntington's. We can prevent cancer...”

But at the time there were many barriers – we had no curative intervention, testing was laborious and expensive, there were not enough genetic counsellors and we thought women would not accept prophylactic bilateral mastectomies. *BRCA1* has not yet made it into medical school curricula. Additionally, geneticists compared this to the Huntington's disease model [where a pathogenic *Htt* variant has 100% penetrance and the disease is uniformly fatal] but, as an oncologist, I disagreed. We *can* cure a lot of cancer *if* we find it early. I absolutely fought against the genetics community, arguing that this is not like Huntington's. We can prevent cancer and it was easy for me to see the path from testing to prevention.

I was fortunate enough to be asked to chair the genetics taskforce at the American Society of Clinical Oncology. The community believed this was going to be transformative for the field. So we started taking family histories of patients, which is the cheapest ‘genetic test’. At a similar time, the Human Genome Project brought about a massive promise and we went from cloning individual genes to precision oncology.

Now, seeing precision oncology as a clinical reality really confirms what physician scientists and interdisciplinary collaboration can do. We understand the problem in the clinic and we can figure out how to solve it in the lab.

**Precision oncology has been successful in targeting oncogenes but therapies for cancers driven by tumor suppressor loss are much more of a challenge**

Yes, the classic divide between a tumor suppressor and an oncogene persists but regulatory networks are interconnected, and the more tools we have to probe these perturbations, the more therapeutic options we open. For example, immunotherapy is more successful in tumors that have abundant neoantigens [likely to arise from deficient DNA repair, such as upon loss of *BRCA1*], something that we were able to fully understand by combining genome sequencing – both germline and cancer – with lab research. Science is exciting because it evolves and allows us to ask new questions.

**In a recent plenary lecture, you talked about studying breast cancer risk variants in Black women, a population that the health system in the US continuously underserves. What are the key lessons?**

The first described families with recurrent *BRCA1* mutations happened to be Ashkenazi Jews, a population that is genetically homogeneous, so it spurred discussions around founder mutations. But I happen to live in a big and diverse city, Chicago, and my hospital serves a very diverse community, so I frequently treat African American patients. The first African American family I identified was so extensive and their phenotype so striking that it prompted a collaboration with Mary-Claire King [the first geneticist to link germline *BRCA1* mutations to familial breast cancer]. This family was just as compelling, but nobody was studying or writing about this population. I thought we would find recurrent mutations. We didn't, but we identified many variants of unknown significance. We tried to apply lessons from a founder population, the Ashkenazi Jews, to the study of one of the most genetically diverse populations, people of African descent. But we stumbled on a huge gap in knowledge and I really wanted to connect the dots. My colleagues thought the variation was ‘contaminating’ the results and wanted to exclude this information. But I said ‘Go on, you can exclude them, but I will continue to study them’ despite how challenging it was. I wanted to understand what was driving the variant enrichment.

African Americans face many structural barriers that exclude them from biomedical research. I thought it was important for us to figure out the risk variants in this population, but also more broadly. If you carry risk variants for triple-negative breast cancer and you don't know it, you may wake up one day at age 35 with a terrible disease, no matter whether you are Black or White. So our goal is to improve patient stratification for risk and for treatment.

**What are the key implications of improved stratification?**

For decades, surgeons would recommend that at-risk women have their breasts removed as a preventative measure. When I set out to do breast cancer research, women became more empowered to advocate for more research and better solutions. As more women advocated for more research, some of the stigma and some of the barriers were being removed. Now women do not have to accept having their breasts removed if they do not want to. Women empowerment is also improving diversity in all areas of biomedical research and healthcare. When we have women and underserved minorities at the table, we gain insights into what we really need to do to achieve progress.

“When we have women and underserved minorities at the table, we gain insights into what we really need to do to achieve progress.”

What I learned from this journey is that we need everybody at the table. We need to talk to both scientists who help us find answers and to diverse patients who help us understand clinical variation. Because African Americans are still distrusting the medical establishment, for valid reasons, we need community engagement to explain how genetics can work for their benefit. A mother who is dying of breast cancer does not want the same to happen to her daughter and, if her cancer is genetic, she can be reassured that we'll continue with the research so that her children won't have to go through what she has gone through. Women are heroines. The field of precision oncology was certainly moved forward by women putting their effort in breast and ovarian cancer research and in advocacy. This has now opened up a whole new area of work for basic research scientists to think about how their work affects society.

“Women are heroines. The field of precision oncology was certainly moved forward by women putting their effort in breast and ovarian cancer research.”

**I found it striking how involved you are in the community. Was this a conscious choice?**

Well, I graduated from medical school in Nigeria for free, so I always felt obligated to give back. When I moved to Chicago unburdened by student loans, I did not need to focus on making money to pay those off. I could focus on research and on serving the community. Also, my research in Nigeria was initially a form of service. But then it got really interesting and it highlighted how much we still needed to do. Unfortunately, blaming patients for their worse outcomes was common and I refused to accept this. I had sufficient funding that allowed me to investigate the science behind the disparities. The MacArthur foundation noticed my work and, after winning the [MacArthur ‘Genius’] fellowship, all bets were off. The work started to get noticed but I was still doing it mostly to pay back.

**Looking back, do you think that your younger self would be surprised by your career path?**

Just this morning, I accompanied my daughter on a nursery school visit for my 8-month-old grandchild and I remembered doing the same visits for my own children. It brought back memories of having intellectual curiosity. I always wanted to do and know more because everything was so interesting. In medical school, we were required to perform a certain number of deliveries and I noticed a lot of women died or had complications during childbirth – and I became interested. So instead of doing the minimum, I ended up delivering 104 babies because I wanted to see if there were patterns to those complications. It made me want to get good at it so I could help more mothers. It exemplified my interest in learning to do better and to be better. Later on, I think I went into research because I thought we could do better in oncology.

**You have been in leadership roles for some time now and are a role model to many. Is there any advice you would like to share with your younger colleagues?**

The way we do things is changing. Nowadays, there's much more acceptance of team science and of new technology. Because of this, the next generation will be even better than us. There is no limit to what science and technology can do. For anyone starting now, my advice is to find what really motivates you and to find a role model and mentor that is accessible to you. Like the late Dr Rowley was to me. Inter-generational mentorship is so important. Because, my goodness, the future is so bright!

**Can you tell us something about you that your colleagues would be surprised to learn?**

I'm an excellent cook and make it a point of cooking at home, ensuring we have dinner as a family every night. I think my colleagues may be surprised to know that.

